# Interface Engineering of TiO_2_ Photoelectrode Coatings Grown by Atomic Layer Deposition on Silicon

**DOI:** 10.1021/acsomega.1c04478

**Published:** 2021-10-07

**Authors:** Jesse Saari, Harri Ali-Löytty, Mari Honkanen, Antti Tukiainen, Kimmo Lahtonen, Mika Valden

**Affiliations:** † Surface Science Group, Faculty of Engineering and Natural Sciences, Tampere University, P.O. Box 692, FI 33014 Tampere, Finland; ‡ Tampere Microscopy Center, Faculty of Engineering and Natural Sciences, Tampere University, P.O. Box 692, FI 33014 Tampere, Finland; § Faculty of Engineering and Natural Sciences, Tampere University, P.O. Box 692, FI 33014 Tampere, Finland

## Abstract

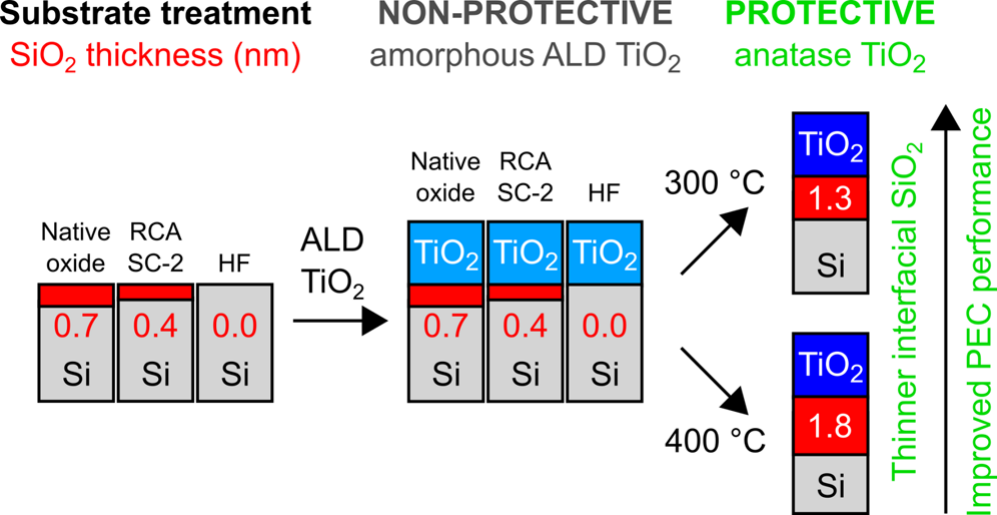

Titanium dioxide (TiO_2_) can protect photoelectrochemical (PEC) devices from corrosion, but the fabrication of high-quality TiO_2_ coatings providing long-term stability has remained challenging. Here, we compare the influence of Si wafer cleaning and postdeposition annealing temperature on the performance of TiO_2_/n^+^-Si photoanodes grown by atomic layer deposition (ALD) using tetrakis(dimethylamido)titanium (TDMAT) and H_2_O as precursors at a growth temperature of 100 °C. We show that removal of native Si oxide before ALD does not improve the TiO_2_ coating performance under alkaline PEC water splitting conditions if excessive postdeposition annealing is needed to induce crystallization. The as-deposited TiO_2_ coatings were amorphous and subject to photocorrosion. However, the TiO_2_ coatings were found to be stable over a time period of 10 h after heat treatment at 400 °C that induced crystallization of amorphous TiO_2_ into anatase TiO_2_. No interfacial Si oxide formed during the ALD growth, but during the heat treatment, the thickness of interfacial Si oxide increased to 1.8 nm for all of the samples. Increasing the ALD growth temperature to 150 °C enabled crystallization at 300 °C, which resulted in reduced growth of interfacial Si oxide followed by a 70 mV improvement in the photocurrent onset potential.

## Introduction

Photoelectrochemical (PEC) solar fuel production from H_2_O and CO_2_ is one of the potential methods for storing solar energy in chemical form as hydrogen and hydrocarbons.^1^ Solar fuel production at a large scale using a PEC reactor requires photoelectrodes that are efficient, chemically stable, and cost-effective. Titanium dioxide (TiO_2_) is a common photocatalyst for solar fuel production but lacks efficiency due to the band gap in the UV range. One viable approach to increase the efficiency is to use semiconductor materials of high-efficiency solar cells, such as Si, GaAs, and GaP, as photoelectrode materials, but because of their intrinsic chemical instability under PEC conditions, a protective coating is required.^2^ Recently, the stability of semiconductor photoelectrodes has been successfully increased by TiO_2_ thin film coatings grown by atomic layer deposition (ALD) using either amorphous^3,4^ or crystalline^5^ TiO_2_. However, the fabrication of high-quality TiO_2_ thin films providing long-term stability to the photoelectrodes has remained challenging and the stability of amorphous TiO_2_ (am.-TiO_2_) has shown to be controversial.^6^

In addition to corrosion protection, the TiO_2_ photoelectrode coating serves as a charge transfer layer between the semiconductor and the catalyst surface of the photoelectrode. Therefore, TiO_2_ photoelectrode protection layers need to be either electrically “leaky” and thick (4–143 nm) am.-TiO_2_,^3^ dielectric, and ultrathin (∼2 nm) to allow the tunneling of charge carriers^4^ or a semiconductor with a favorable band alignment with respect to the substrate semiconductor and the energy level of a chemical reaction.^5^ The electrically “leaky” TiO_2_ has the ability to conduct holes due to midgap defect states in the electronic structure of am.-TiO_2_.^3,7^ However, the stability of am.-TiO_2_ without additional co-catalyst has remained controversial.^8^ In contrast to am.-TiO_2_, crystalline TiO_2_ is thermodynamically stable under alkaline water splitting conditions.^6^ Furthermore, with sufficient contact to catalyst material, the crystalline n-type TiO_2_ coating can also behave as a charge transfer layer and conduct electrons from the catalyst–electrolyte interface to the semiconductor substrate in a photoanode and vice versa in a photocathode.^9,10^ In our recent studies, we have reported means to thermally modify the defect structure of ALD-grown am.-TiO_2_ thin film under oxidative^6^ and reductive^11^ conditions. Based on our research, annealing in air at 500 °C results in stable and photocatalytically active crystalline TiO_2_.^6^ However, compared to the oxidation a heat treatment in reductive ultrahigh vacuum (UHV) at 500 °C can retain the amorphous phase for TiO_2_ and enhance the stability due to the formation of O^–^ species via electron transfer from O to Ti.^11^

Atomic layer deposition providing good controllability, uniformity, and conformality can be used to fabricate high-quality and pinhole-free TiO_2_ photoelectrode protection layers.^4,12,13^ The choices of precursors and process conditions affect the TiO_2_ phase structure. ALD of crystalline TiO_2_ has been reported using TiCl_4_ (at 200 °C) or TTIP (at 250 °C) as titanium precursors and H_2_O or O_3_ as oxygen sources, respectively.^14,15^ The growth of TiO_2_ using more volatile TDMAT and H_2_O allows ALD at growth temperatures as low as 50 °C, which enables growth on sensitive materials.^16^ However, based on our knowledge, there are no reports on thermal ALD from TDMAT and H_2_O between 100 and 200 °C that results in a fully crystalline TiO_2_ film without additional heat treatment.^6,17^ Growth at higher temperatures could result in crystalline TiO_2_, but the thermal decomposition of TDMAT challenges the self-limiting ALD process.^18−20^

Substrate pretreatment, interface engineering of a TiO_2_/semiconductor heterojunction, and the morphology of TiO_2_ are the key factors affecting charge carrier transport of the heterojunction and thus the performance of photoelectrodes.^7,14,21,22^ For example, on Si-based electrodes, the growth of a resistive interfacial silicon oxide layer at the TiO_2_/Si interface can prevent the charge transfer.^7,23^ According to Scheuermann et al., less than 2 nm thick SiO_2_ has no substantial effect on conductivity, but the performance of the photoanode could be remarkably improved by preparing a TiO_2_/Si heterojunction with a less than 1 nm thick interfacial silicon oxide.^23^ Cho et al. reported the effect of the substrate surface energy on the grain size of as-deposited ALD TiO_2_ films grown from TTIP and O_3_ on SiO_2_, Al_2_O_3_, HfO_2_, and Pt substrates.^14^ The deposition on a high-surface-energy substrate can lead to large anatase grains (2–3 μm) due to the higher interfacial energy between TiO_2_ and the substrate, which decreases the number of crystal nuclei on the surface.^14^ Furthermore, Pore et al. were able to prepare much larger explosively crystallized anatase TiO_2_ grains with a width of several tens of microns by postannealing amorphous Ti–Nb–O or Ti–Ta–O mixed oxide films.^24^ The large anatase grain size accompanied with small grain boundary volume is reported to improve the thermal stability and photocatalytic activity of TiO_2_ thin films,^24,25^ which are also desired features for protective photoelectrode coatings since corrosion reactions are often initiated at grain boundaries.

The surface termination of the Si substrate depends on the surface treatment and can strongly influence the ALD growth that is essentially a surface-mediated process.^21,22,26−28^ Prior to ALD, native SiO_2_ can be removed from the surface by HF treatment, which terminates the Si surface by Si–H bonds.^26^ The hydrogen-terminated Si surface is hydrophobic, lowering the initial ALD growth rate of TiO_2_ due to the slower adsorption of ALD precursor molecules on the Si surface.^22,26^ For example, McDonnell et al. reported 185 times greater TiO_2_ deposition rate on an oxide-terminated Si compared to a H-terminated surface using TiCl_4_ and H_2_O.^28^ Devloo-Casier et al. reported that HF treatment of Si changed the growth mode from layer by layer to island growth for HfO_2_ using tetrakis(ethylmethylamino)hafnium and H_2_O.^27^ In contrast to the HF treatment, boiling in HCl–H_2_O_2_–H_2_O (RCA SC-2 treatment^29^) leads to a hydrophilic surface due to the high density of surface hydroxyl groups,^30,31^ which are reported to influence the crystallization and grain size of ALD TiO_2_ grown from TiCl_4_ and H_2_O precursors.^21,22^ Therefore, the substrate pretreatment is of primary importance especially to the fabrication of ultrathin pinhole-free tunnel oxides and 2D materials for the semiconductor technology^13^ but can be also utilized in area-selective growth.^28^

This work examines the influence of silicon wafer pretreatment (1) on the initial ALD TiO_2_ (0–2 nm) growth; (2) on the TiO_2_/Si interface composition; and (3) on the performance of TiO_2_ (30 nm) protective coating on Si under photoelectrochemical (PEC) water splitting conditions. ALD TiO_2_ thin films were grown from TDMAT and H_2_O precursors at 100 °C (1) on native Si oxide; (2) on oxide-free Si surface after exposing the Si wafer to dilute HF solution; and (3) on chemical Si oxide that forms on an HF-dipped Si wafer surface during boiling in HCl–H_2_O_2_–H_2_O. The as-deposited TiO_2_ thin films were amorphous and dissolved under alkaline PEC conditions. The stability of the TiO_2_ thin film over a time period of >10 h under PEC conditions was obtained after heat treatment in air at 400 °C that induced crystallization of am.-TiO_2_ into anatase TiO_2_. Nondestructive X-ray photoelectron spectroscopy analysis was applied to quantitatively analyze the morphology of TiO_2_ (2 nm)/SiO_2_/Si heterostructures and revealed that no interfacial Si oxide formed during the ALD growth, but during the heat treatment, the thickness of interfacial Si oxide increased to 1.8 ± 0.1 nm for all of the samples. By increasing the growth temperature from 100 to 150 °C, the crystallization temperature can be decreased from ∼400 to 300 °C,^17^ which limits the growth of interfacial Si oxide and is shown to result in more significant improvement in the PEC performance compared to the wafer pretreatments.

It is evident from the results shown here that growing a high-quality ALD TiO_2_ thin film on Si wafer depends on how the Si surface is cleaned, albeit the choice of the cleaning method affected only little the final structure and properties of the 30 nm thick TiO_2_ thin film as a photoelectrode coating on Si. Further improvement in the quality of the TiO_2_/Si photoelectrode would require either the development of the ALD growth process itself or the postgrowth heat treatment of the as-deposited TiO_2_ thin film to result in a crystalline low-defect TiO_2_ structure at a lower temperature and thereby avoiding the formation of interfacial Si oxide that is detrimental to the charge transfer. These properties are not sensitive to the doping of Si substrate, and therefore, our results obtained using degenerately doped n^+^-Si as the substrate are applicable to Si-based photoelectrodes in general.

## Materials and Methods

### Substrates

In the experiments, the degenerately Sb-doped (resistivity 0.008–0.02 Ω cm) n^+^-Si(100) wafers from SIEGERT WAFER GmbH (Germany) were used as substrates. The use of degenerately doped Si substrates allowed the investigation of photogenerated charge carriers within TiO_2_ coating only, while Si substrate served as a conductor. Prior to atomic layer deposition of TiO_2_, some of the substrates were treated with hydrofluoric acid (HF), some with HF followed by RCA SC-2 treatment (Radio Corporation of America standard clean 2),^29^ and some of the substrates having a thin native oxide (SiO_2_) layer were used as received from the wafer vendor. In the HF treatment, the Si wafer was immersed in 2.5% hydrofluoric acid (HF) for 10 s, then rinsed in two different deionized water (DI-H_2_O) containers, in the first one for 3 s and in the second for 10 s. After this, the samples were blown dry with nitrogen. The HF treatment etches the native oxide layer, resulting in a H-terminated hydrophobic Si surface.^21,31^ In the RCA SC-2 treatment, i.e., chemical oxidation, the Si wafer was soaked in a 6:1:1 H_2_O/30% H_2_O_2_/37% HCl solution at 70–75 °C for 10 min.^29^ After the treatment, the wafer was rinsed with DI-H_2_O and blown dry with nitrogen. This treatment produces a silicon wafer with a thin silicon oxide layer that is hydrophilic due to the high density of hydroxyl groups (−OH) on the surface.^30,31^ [Caution: HF is highly corrosive and requires the use of Teflon, rather than glassware, and can easily penetrate the skin, bond with Ca^2+^, and cause nerve damage. As such, even a small exposure (e.g., 2–10% of the body) can be fatal. Proper training is required before handling or working with HF, and appropriate personal protection equipment should be worn at all times when carrying out these sample preparations.]

### Water Contact Angle (CA) Measurements

The water contact angle measurements were performed using an Attension Theta contact angle meter equipped with an Automatic Single Liquid Dispenser. The DI-H_2_O drop size used for the experiments was 5.0 ± 0.5 μL. The drop was stroked on the surface and given to stabilize for 2–3 s. The right and left contact angles were recorded for 10 s (15 frames per second), and the contact angle was determined as an average of the right and left contact angles.

### Atomic Layer Deposition (ALD)

ALD of TiO_2_ was carried out using a Picosun Sunale ALD R-200 Advanced reactor and tetrakis(dimethylamido)titanium(IV) (Ti(N(CH_3_)_2_)_4_, TDMAT, electronic grade >99.999%, Sigma-Aldrich) and deionized water as precursors. To reach the proper TDMAT precursor vapor pressure, the bubbler was heated to 76 °C, and to prevent condensation of the precursor gas, the delivery line was heated to 85 °C. The water bubbler was sustained at 18 °C by a Peltier element for stability control. Argon (99.9999%, Oy AGA Ab, Finland) was used as a carrier gas. During the ALD, the Si substrates were held at 100 °C. A lower deposition temperature was chosen to get more stoichiometric TiO_2_ and to hinder the growth of the resistive interfacial silicon oxide layer. The thickness of the TiO_2_ (480 ALD cycles) film was measured by ellipsometry and was 30 nm (Rudolph Auto EL III Ellipsometer, Rudolph Research Analytical). Based on the calculated growth rate, the number of ALD cycles for studying the initial growth was selected to be 1, 8 (5 Å), and 32 (2 nm). At a 150 °C growth temperature, 636 ALD cycles were required for a 30 nm thick TiO_2_ thin film.^17^

### Heat Treatment

The post-treatment for the TiO_2_/Si samples was performed in air by placing the samples directly into a preheated tube furnace for 45 min. After the heat treatment, the samples were removed from the tube furnace and let to cool down freely. The heat treatment temperature for TiO_2_ grown at 100 °C was optimized to yield the maximum photocurrent for H_2_O oxidation (Figure S4). The heat treatment for the TiO_2_ grown at 150 °C was decided based on the crystallization temperature.^17^

### X-ray Photoelectron Spectroscopy (XPS)

The chemical composition and thin film morphology of sample surfaces were analyzed with X-ray photoelectron spectroscopy (XPS). Nonmonochromatic Al Kα (*h*ν = 1486.5 eV) X-ray was used as an excitation source. The core-level XP spectra were analyzed by the least-squares fitting of Gaussian–Lorentzian lineshapes and using a Shirley-type background. The binding energy scale of the XP spectra was calibrated by fixing the Si^0^ 2p_3/2_ peak to 99.3 eV. CasaXPS version 2.3.22 PR1.0. was used as the analysis software, and the Scofield photoionization cross sections were used as relative sensitivity factors. The quantitative analysis of the TiO_2_/SiO_2_/Si heterostructure morphology was based on the attenuation of photoelectron signal in solid material according to the Beer–Lambert law and is described in detail in the Supporting Information.

### Scanning Electron Microscopy (SEM)

The surface morphology of Si substrates after different pretreatments was studied by scanning electron microscopy (SEM, Zeiss Ultra 55, Carl Zeiss Microscopy GmbH). The SEM images were measured using in-lens mode with a working distance of 2.3 mm, an electron high tension (EHT) of 1.00 kV, and an aperture size of 30.00 μm.

### Electron Backscatter Diffraction (EBSD)

The electron backscatter diffraction (EBSD) analysis was carried out using SEM (Zeiss Ultra Plus, Carl Zeiss Microscopy GmbH) equipped with an EBSD system (Symmetry, Oxford Instruments). The EBSD maps were collected using a 70° sample tilt, an EHT of 10 kV, an aperture size of 120 μm, and a step size of 0.1 μm. Here, pattern quality, i.e., band contrast (BC), maps were presented. The BC map represents the quality of the Kikuchi pattern for each measurement pixel; bright signifies good pattern quality and black poor quality such as in grain boundaries.

### Grazing-Incidence X-ray Diffraction (GIXRD)

The phase structures of the samples were obtained via grazing-incidence X-ray diffraction (GIXRD, PANalytical X’Pert^3^ MRD diffractometer) with Cu Kα radiation (λ = 1.5406 Å, *h*ν = 8.05 keV) and 45 kV and 40 mA cathode voltage and current, respectively. The samples were scanned in 2θ between 20 and 52° using a grazing-incidence angle Ω = 0.3°. Background was removed from each of the scans to allow easier comparison of the measured curves.

### Photoelectrochemical (PEC) Analysis

The photoelectrochemical performance was studied in a homemade PEC cell using three-electrode configuration with an Ag/AgCl reference electrode, Pt counter electrode, and TiO_2_/Si sample as the working electrode following the procedure described in detail in our previous work.^6^ The measurement was controlled by an Autolab PGSTAT12 potentiostat (Metrohm AG). An aqueous solution of 1 M NaOH (pH 13.6) was used as an electrolyte. Simulated solar spectrum was produced with a HAL-C100 solar simulator (Asahi Spectra Co., Ltd., JIS Class A at 400–1100 nm with AM1.5G filter), and the intensity was adjusted to 1.00 Sun using a 1 sun checker (model CS-30, Asahi Spectra Co., Ltd.).

## Results and Discussion

**Figure 1:**
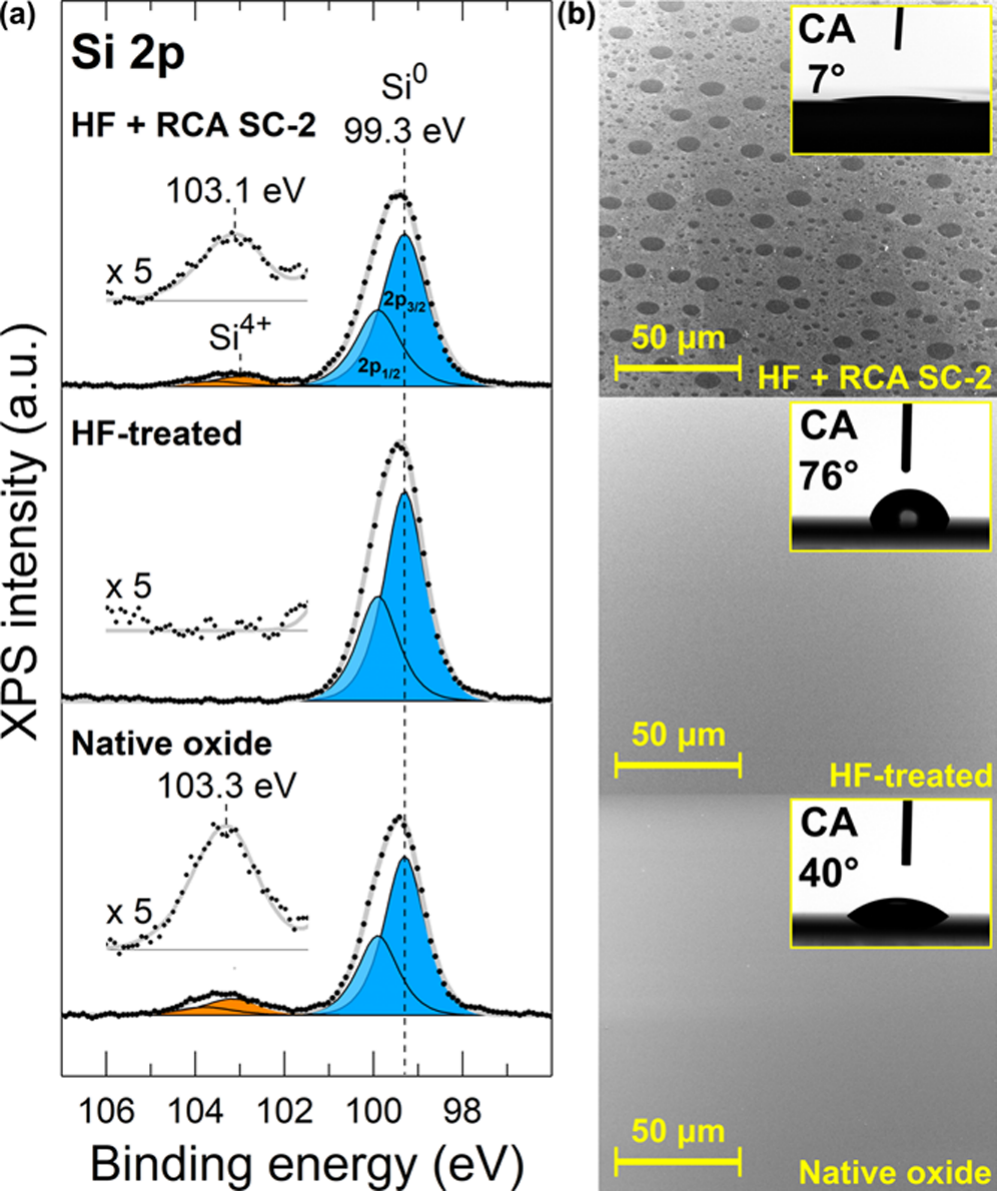
(a) XP Si 2p spectra and (b) SEM images and CA measurements for the native oxide, HF-treated, and HF + RCA SC-2-treated Si(100) substrates.

Figure 1 shows Si 2p XP spectra and SEM images together with water contact angle measurements for the Si(100) surfaces after different surface treatments, i.e., the surface condition prior to the atomic layer deposition of TiO_2_. The XP spectra of Si 2p transition show a strong doublet peak with Si 2p_3/2_ at 99.3 eV corresponding to elemental Si from the Si substrate. Other peaks are observed at 103.3 eV for the native oxide and at 103.1 eV for the chemical oxide that are both assigned to oxidized Si, mainly Si^4+^ oxide, with a slight deviation in the chemical environment between the two oxides. In contrast, Si oxide was not detected on the HF-treated sample, which confirms the temporal passivation of Si surface against oxidation. The doublet separation of 0.60 eV was applied in peak fitting for all of the chemical states in Si 2p.

SEM images and water contact angles presented in Figure 1b reveal the disparity in surface morphology and hydrophilicity between different surface treatments. The surface with native oxide is flat, and the water contact angle is rather low (40°) in accordance with the literature.^31,32^ HF treatment results in flat surface morphology and a large CA (76°), indicating the hydrophobic behavior caused by the H-terminated Si surface.^31−33^ Thus, both the native oxide and HF-treated Si(100) surfaces were flat and homogeneous albeit chemically different. In contrast, the RCA SC-2 process results in a distinct nonhomogeneous surface morphology that is strongly hydrophilic with CA of 7°. The surface is covered with round voids that have the size of up to 10 μm in diameter. Such a nonhomogeneous surface morphology can be linked to the H_2_ bubble formation that takes place vigorously in the freshly prepared SC-2 solution upon the decomposition of H_2_O_2_. Gas bubbles may have adhered to the surface limiting the surface reactions and thus led to void formation. Similar void formation has been reported to result from cleaning in Fe-contaminated alkaline SC-1 solution (NH_4_OH/H_2_O_2_/H_2_O), where Fe catalyzes H_2_O_2_ decomposition^34^ but rarely upon cleaning in acidic SC-2. Bubble-induced void formation can be effectively mitigated by the implementation of sonication to the RCA cleaning procedure.^35^ Despite the nonhomogeneous surface morphology, the treatment was found reproducible and served as an interesting substrate for the ALD TiO_2_ thin films. The hydrophilicity is attributed to the high density of hydroxyl groups on the surface.^30,31^ Compared to the native oxide, the Si^4+^ 2p peak in the RCA SC-2-treated Si appeared to be 0.2 eV closer to the Si^0^ peak that is now supported with the difference in the surface termination and with the nonhomogeneous oxide layer. The lower binding energy can also indicate the presence of some Si suboxides (Si^3+^–Si^1+^) reported to exist in a chemically grown Si oxide.^36^ The amount of adventitious carbon was similar (4–6 at. %) for all of the surface treatments (Table S1), and no metal impurities were detected.

**Figure 2:**
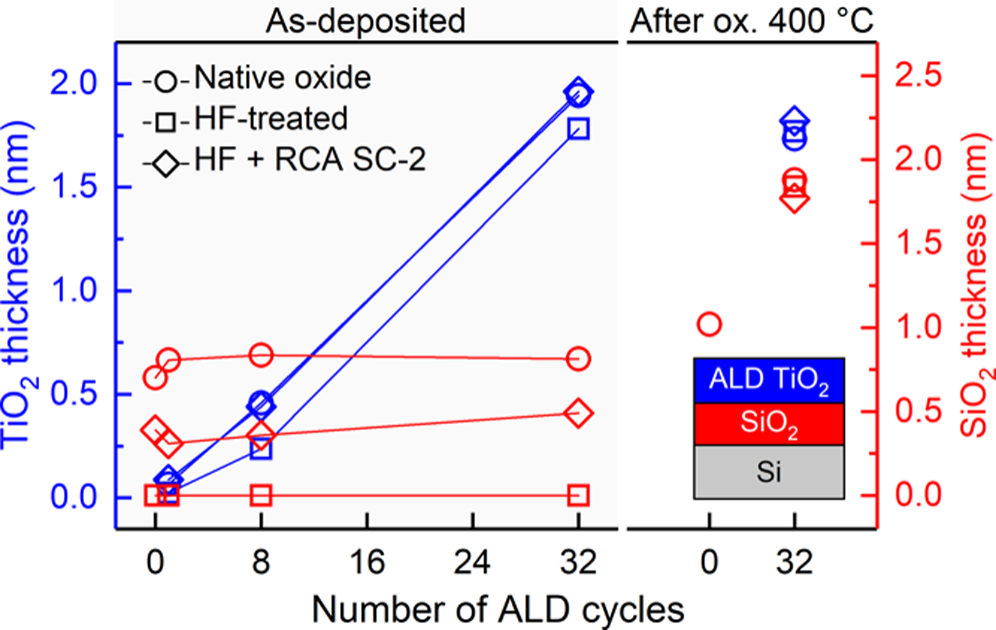
Thicknesses of TiO_2_ surface layer and interfacial SiO_2_ as a function of ALD TiO_2_ cycles and after oxidation in air at 400 °C for different surface treatments.

To study the influence of surface treatment on the initial ALD TiO_2_ growth and on the formation of interfacial Si oxide, surface analysis by XPS was performed as a function of ALD TiO_2_ cycles and after oxidation in air at 400 °C as shown in Figure 2. The growth rate of ALD TiO_2_ as determined by electron spectroscopy was found to follow a linear trend (0.06 ± 0.01 nm/cycle) on oxidized surfaces, i.e., the native oxide and HF + RCA SC-2 surfaces, in good agreement with the average growth per cycle (GPC = 0.063 ± 0.003 nm/cycle) determined for a 30 nm thick ALD TiO_2_ layer using ellipsometry. In contrast, the initial growth on oxide-free Si substrate (HF-treated) was strongly hindered up to eight ALD cycles after which the growth rate continued at the same rate as with the other Si surfaces. Thus, the HF treatment did not change the growth from layer-by-layer to island mode,^27^ which would have resulted in an increase in the apparent growth rate. This inhibition period on the oxide-free surface resulted effectively in a 0.2 nm smaller TiO_2_ film thicknesses compared to oxide surfaces when total TiO_2_ thickness exceeded 0.2 nm (∼1 monolayer of TiO_2_). The initial growth rate is lower on H-terminated Si and higher on SiO_2_ surfaces due to the enhanced precursor adsorption and surface reactions with hydroxyl groups on the surface.^26^

The thickness of the interfacial Si oxide layer did not change during the ALD process. Most significantly, the TiO_2_/Si interface for the HF-treated surface remained free from Si oxides, which is largely due to the low growth temperature of 100 °C. A similar result was observed by Methaapanon et al.^26^ The thicknesses of interfacial Si oxide in the native oxide and HF + RCA SC-2 -treated samples were 0.8 ± 0.1 and 0.4 ± 0.1 nm, respectively. The chemical oxide that forms in the rapid HF + RCA SC-2 treatment was not fully developed and is expected to reach the thickness of native oxide with time when exposed to air. The Si^4+^ 2p binding energy for the interfacial Si oxide was shifted by −0.4 eV compared to the Si^4+^ 2p at the surface (Figure S1). The formation of an interfacial compound might induce such a binding energy shift. However, based on our nondestructive angle-resolved XPS analysis, the Ti–O–Si interface width was only 0.4 nm (∼1 monolayer of SiO_2_ or TiO_2_), which does not support substantial mixing of TiO_2_ and Si oxide (Figure S2).^37^ More specifically, only Ti^4+^ and no suboxides of Ti, i.e., Ti^1–3+^, were present since the normalized Ti 2p spectra (Figure S2a) recorded at different electron emission angles were identical. The Si^4+^ 2p binding energy shift was therefore assigned to the TiO_2_ overlayer-induced change in the chemical environment.^26^ This contradicts the work by Dwivedi et al. who reported mixed oxide (SiO*_x_* + TiO*_x_*) interfacial layer formation with Ti^2+^ for ALD TiO_2_ grown on HF-treated Si at 100 °C from TiCl_4_ and H_2_O.^38^

The heat treatment in air at 400 °C did not affect the thickness of TiO_2_ overlayer. However, the thickness of the interfacial Si oxide layer was increased to 1.8 ± 0.1 nm for all of the samples despite the difference before the heat treatment. No substantial mixing of TiO_2_ with Si oxide was observed, but the width of the Ti–O–Si interface evidenced a slight increase from 0.4 nm (as-deposited) to 0.6 nm (400 °C) as analyzed in more detail in the case of HF + RCA SC-2-treated substrate (Figure S2). In addition, the thickness of the interfacial Si oxide was observed to increase linearly with heat treatment temperature from 200 to 550 °C (Figure S3). Interestingly, the same heat treatment induced only minor oxide growth on the bare Si substrate, i.e., 0 ALD cycles in Figure 2. We suggest that this apparent discrepancy is due to the higher catalytic activity of the TiO_2_ surface to dissociate O_2_, i.e., the initial step of oxidation, compared to SiO_2_ surface.^39,40^ This so-called catalytic effect on the interfacial SiO_2_ formation has been observed also for other high-κ metal oxide materials on silicon.^41^ The heat treatment at 400 °C did not affect the Si^4+^ 2p binding energy, and the morphology analysis did not support intermixing of SiO_2_ with TiO_2_ (Figure S2).

The heat treatment temperature was optimized in terms of the performance of the 30 nm thick ALD TiO_2_ thin film grown at 100 °C on n^+^-Si to act as a photocatalyst for water oxidation. We note that in the experiment, only TiO_2_ contributes to the photocurrent, while the degenerately doped n^+^-Si substrate serves as a conductor. The maximum photocurrent was measured after heat treatment at 400 °C (Figure S4). For higher heat treatment temperatures, the photocurrent decreased slightly up to 500 °C followed by a drastic drop at 550 °C. The decrease in photocurrent is attributed to the increase of the resistive interfacial Si oxide layer thickness above the tunneling limit, which was estimated to be ∼3 nm based on an experiment performed using ALD TiO_2_ (2 nm)/Si model system (Figure S3). For temperatures below 400 °C, the TiO_2_ thin film was not stable under PEC conditions. Previously, we have shown that a high temperature is required to convert chemically unstable amorphous ALD TiO_2_ into stable crystalline TiO_2_.^6^ Therefore, it can be concluded that further improvement in the performance of ALD TiO_2_/Si photoelectrode would require the development of ALD growth parameters that enables fabrication of crystalline TiO_2_ at a lower temperature where resistive interfacial SiO_2_ does not form. To constrain the thickness of the interfacial Si oxide below 1 nm limit for improved charge transfer as was suggested by Scheuermann et al.,^23^ the oxidation temperature should not exceed 250 °C in the case of studied 45 min heat treatment time (Figure S3).

**Figure 3:**
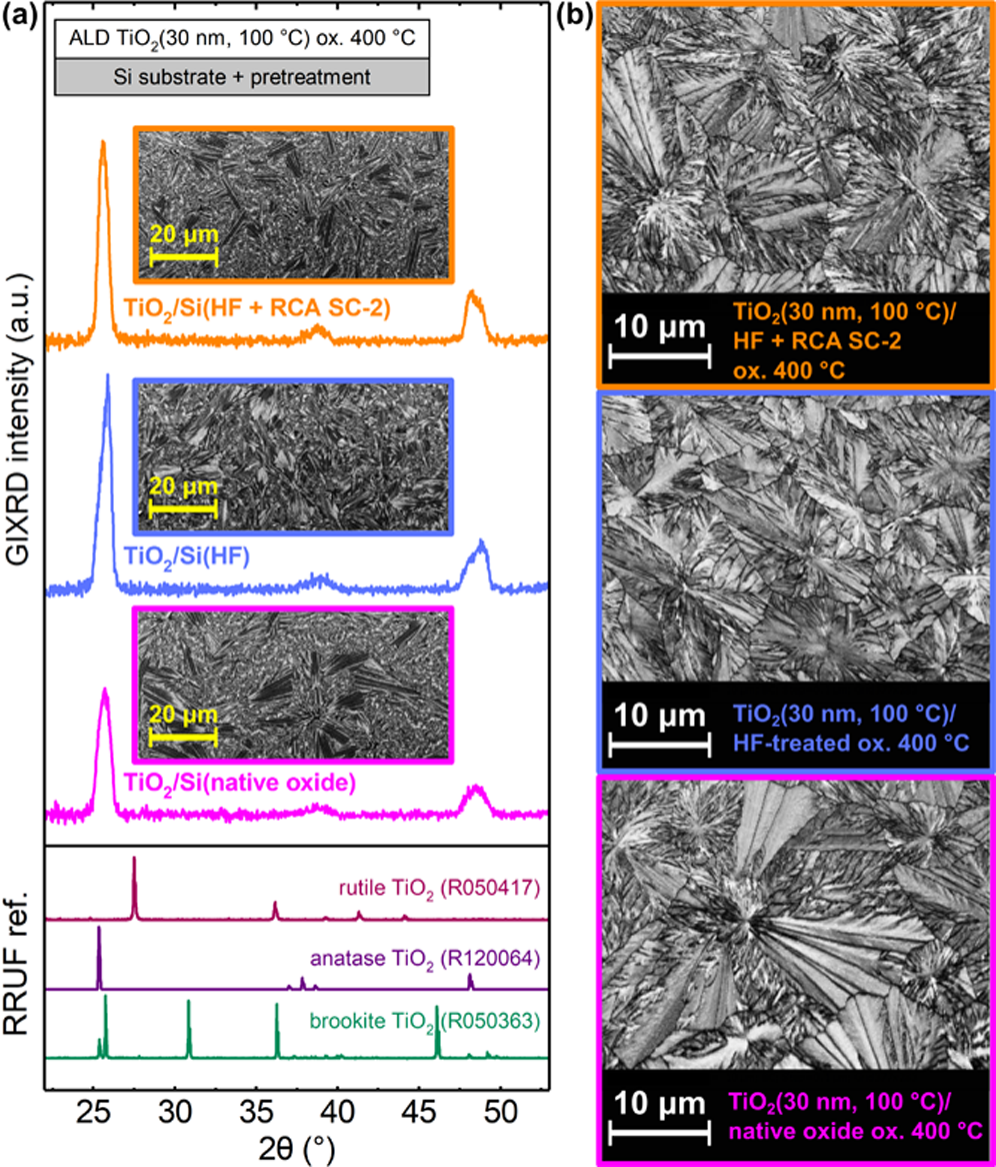
(a) GIXRD patterns and SEM images (insets) and (b) BC maps of 30 nm thick ALD TiO_2_ films after oxidation at 400 °C for as-deposited am.-TiO_2_ grown on native oxide, HF-treated, and HF + RCA SC-2-treated Si(100) substrates. The XRD references are from RRUF database.^42^

Figure 3 shows GIXRD patterns and BC maps for 30 nm thick ALD TiO_2_ films after oxidation at 400 °C for different surface treatments. The GIXRD patterns for oxidized ALD TiO_2_ thin films corresponded to anatase TiO_2_ for all of the surface treatments and showed no features of other phases of TiO_2_ such as rutile or brookite. Small differences in the anatase XRD peak shapes were observed, suggesting differences in anatase crystal morphology, crystal anisotropy, or thin film stress. The BC maps in Figure 3b show fern leaf-like grains for all anatase TiO_2_ thin films. Similar grain morphology has been reported to be typical for explosively crystallized thin films.^14,24^ The determination of crystallographic orientation and local misorientation maps from the EBSD data was very challenging due to stretched and distorted Kikuchi patterns. However, the anatase thin film on native oxide had the largest crystals with the lateral size of nearly 20 μm. Thus, the largest crystals were over 500 times larger than the film thickness. Moreover, the anatase crystals on HF and HF + RCA SC-2-treated Si appeared to be exceptionally large (about 10 μm) but still smaller than the largest grains on the native oxide substrate.

In addition to the precursor adsorption and the initial ALD growth rate, the differences in the water contact angle are related to differences in the substrate surface energy, which was reported to affect crystallization and the grain size of TiO_2_ during the ALD growth.^14,22^ Larger grains are observed on substrates with higher surface energy that corresponds with a lower contact angle.^14^ Our results suggest that a high substrate surface energy could also mediate the growth of large grains upon postgrowth heat treatment. However, based on the substrate surface energy, HF + RCA SC-2-treated Si should have the largest grains, but the nonhomogeneous and morphologically uneven surface can provide increased number of nucleation sites for crystallization compared to the flat native oxide SiO_2_.

The XPS results presented in Table S2 show similar elemental concentrations for all of the as-deposited am.-TiO_2_ films and also some nitrogen traces (1.0–1.3 at. %) that are most likely dimethylamino fragments from the dissociative adsorption of TDMAT or dimethylamine readsorbing on certain sites of the growing film.^43^ The surface concentration of N was found to decrease to 0.3–0.5 at. % upon heat treatment at 400 °C. This implies that nitrogen traces in am.-TiO_2_ drive the phase stabilization toward anatase instead of rutile, as discussed in our previous article.^6^ TiO_2_ films grown at 200 °C were amorphous, contained less nitrogen traces, and crystallized into rutile in a similar heat treatment. This raises a question if the nitrogen traces mediate the explosive crystallization serving nucleation sites for the large anatase crystals. According to Hukari et al., nitrogen in am.-TiO_2_ inhibits the crystallization and raises the nucleation temperature.^44^ Indeed, we have observed crystallization temperature to depend on the ALD growth temperature,^17^ which in return affects the amount of TDMAT traces in the thin film.^18^ Based on this, we conclude that the explosive crystallization and the large grain size are likely caused by the nitrogen-containing fragments of the TDMAT precursor.

**Figure 4:**
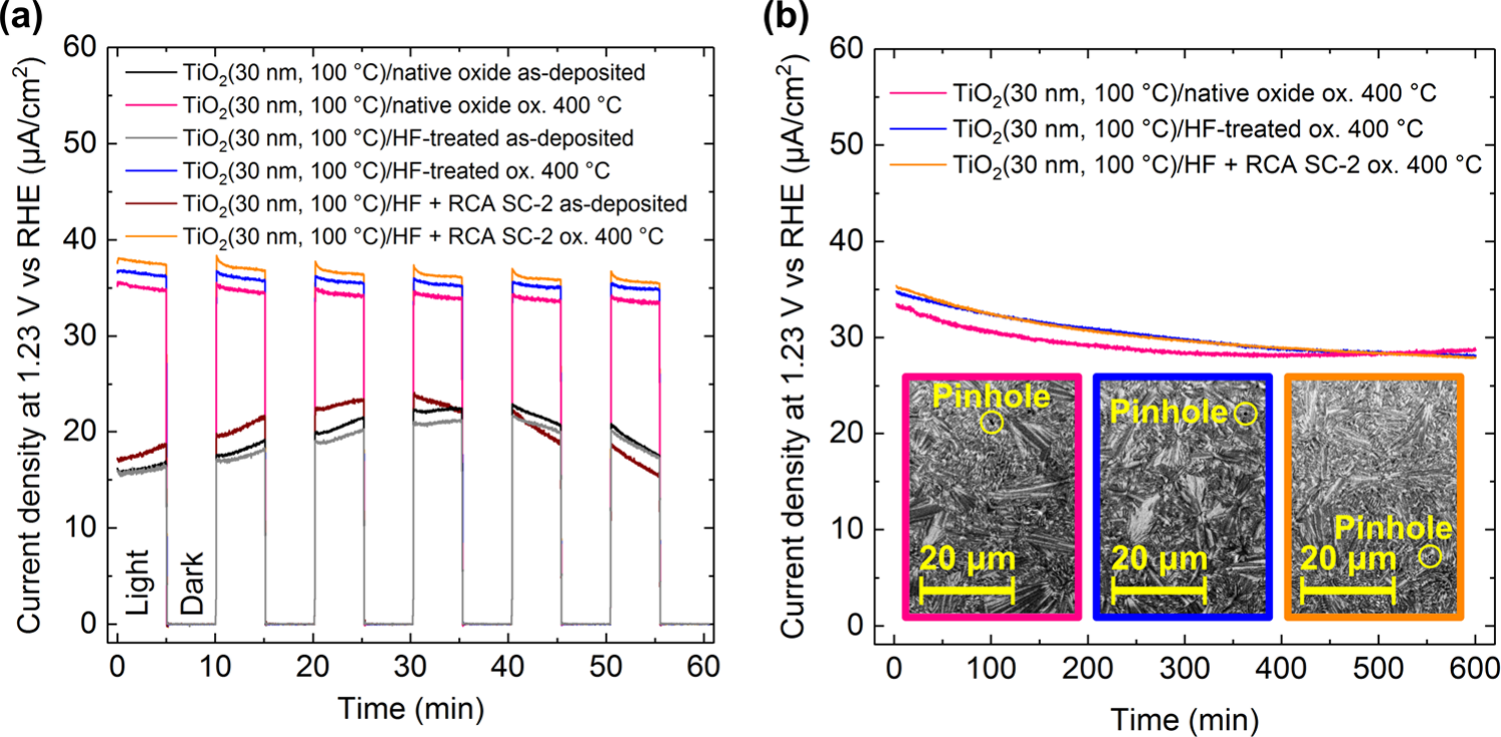
(a) Chopped light (1 Sun) constant potential amperometry measurement in 1 M NaOH for as-deposited (amorphous) TiO_2_ and at 400 °C oxidized (anatase) TiO_2_ thin films on n^+^-Si substrates with different surface treatments; (b) 10 h stability tests for the ox. 400 °C samples under 1 Sun. The insets in (b) are SEM images of the surfaces after the 10 h stability test.

Figure 4a shows chopped light constant potential amperometry measurement results for TiO_2_/n^+^-Si photoelectrodes before and after oxidation at 400 °C for different surface treatments. The measurement reveals photoelectrochemical performance of TiO_2_/n^+^-Si photoelectrodes in terms of stability and photocatalytic activity for solar water oxidation. Regardless of the Si surface treatment, the as-deposited amorphous TiO_2_ films evidenced highly unstable photocurrent: first, the photocurrent increases until after 30–40 min, it starts to decline steadily. For similar TiO_2_/Si samples, we have shown that this photocurrent trend leads to complete dissolution of TiO_2_ coating in <10 h.^11^ Indeed, after the stability test, the dissolution of amorphous TiO_2_ coatings was verified for all of the surface treatment by visual inspection of photoelectrodes showing a color change from yellowish intact coating to gray as the electrolyte had reached the Si substrate (Figure S5). Better understanding of degradation mechanism calls for more detailed studies and is beyond the scope of this work. In contrast, anatase TiO_2_ films for all Si surface treatments show a stable photocurrent of ∼30 μA/cm^2^ for 10 h (Figure 4b). SEM analysis of anatase TiO_2_ samples after the stability test revealed only a few pinholes in the films as shown in the insets in Figures 4b and S6. Thus, despite the minor differences in 30 nm thick anatase TiO_2_ thin film morphologies, they all serve as protection layers for Si substrate that would otherwise dissolve in NaOH.^35^ Therefore, it can be concluded that Si surface treatment had little effect on the PEC performance of TiO_2_/n^+^-Si photoelectrodes. However, we note that this result is not generic to all ALD processes. For example, surface treatment-induced change in the growth mode from layer-by-layer to island growth results more probably in nonprotective ALD coating. Therefore, optimization of surface treatment is required for each photoelectrode system.

The current–voltage (*I*–*V*) characteristics measured under simulated solar light after crystallization under oxidative conditions (anatase TiO_2_) showed a sluggish increase in photocurrent and a low photocurrent at 1.23 V (Figure S7) compared to rutile TiO_2_ fabricated using similar synthesis but at a higher ALD growth temperature of 200 °C.^6^ The sluggish increase in photocurrent is an indication of slow kinetics and high degree of recombination, which we assign to the N traces in the anatase TiO_2_ thin films. The difference in the photocurrent at 1.23 V, on the other hand, is also affected by the difference in band gap energies between anatase and rutile TiO_2_, and by the difference in grain size.^45^ Rutile TiO_2_ has a smaller band gap and therefore absorbs a larger fraction of solar spectrum, which makes it a better photocatalyst. Anatase TiO_2_ with a larger band gap is more transparent to solar light and is therefore better suited as a protective window material for solar cells.^5^

Recently, we showed that amorphous-to-anatase phase-transition temperature for the TDMAT and H_2_O ALD TiO_2_ process can be decreased from ∼400 to 300 °C by increasing the growth temperature from 100 to 150 °C.^17^ Next, we leverage this finding to improve the performance of the ALD TiO_2_/n^+^-Si photoelectrode.

**Figure 5:**
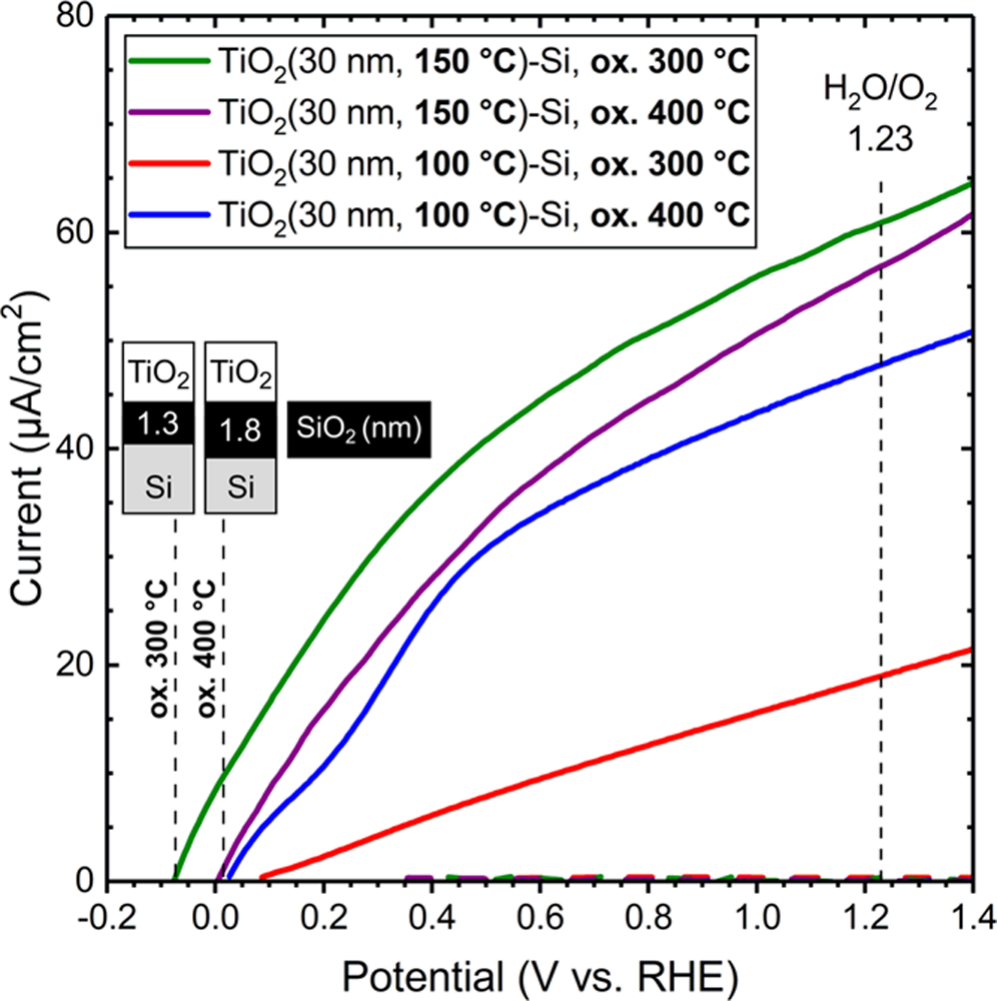
Current–voltage characteristics in the dark (dashed lines) and under simulated solar illumination (solid lines) measured in 1 M NaOH by linear sweep voltammetry for ALD TiO_2_ (30 nm) thin films grown at 100 and 150 °C on n^+^-Si with native oxide and subsequently oxidized at 300 and 400 °C. SiO_2_ layer morphologies after oxidation at 300 and 400 °C are illustrated as insets.

Figure 5 shows the characteristics for ALD TiO_2_ (30 nm) thin films grown at 100 and 150 °C and subsequently oxidized at 300 and 400 °C. For the amorphous TiO_2_ (growth temperature of 100 °C, oxidation at 300 °C), the photocurrent generation is strongly limited due to the high degree of defect states and fast recombination of photogenerated charge carriers. The ALD TiO_2_ thin films grown at 150 °C depict steeper photocurrent onsets after both oxidation temperatures compared to the TiO_2_ thin film grown at 100 °C and oxidized at 400 °C. Interestingly, the photocurrent slope decreases temporarily at 0.14 V for the TiO_2_ thin film grown at 100 °C but not for the thin films grown at 150 °C. We suggest that this feature is due to the difference in the degree of nitrogen-bearing TDMAT traces in the anatase TiO_2_ that affect photocurrent kinetics. Most strikingly, the photocurrent onset potentials (+0.02 ± 0.02 V) were found similar for the TiO_2_ thin films oxidize at 400 °C, whereas the photocurrent onset potential was decreased to −0.07 V for the TiO_2_ thin film grown at 150 °C and oxidized at 300 °C. The decrease in photocurrent onset potential followed an increase in the photocurrent at the water redox potential of 1.23 V. The improved performance can be assigned to the thinner interfacial Si oxide layer that forms at a lower oxidation temperature (i.e., 1.3 nm; cf. Figure S3).

The thickness of the interfacial Si oxide layer is directly associated with the photovoltage loss in oxide-protected water splitting anodes and should be minimized for improved efficiency.^46,47^ Here, we have demonstrated that compared to the postdeposition annealing (PDA) temperature, the silicon wafer cleaning had only little effect on the ALD TiO_2_/n^+^-Si photoelectrode performance. Currently, the performance is not limited by the native oxide on Si wafers but by the excessive PDA temperature that is required to form crystalline low defect protective TiO_2_ coating. We have found earlier that the required minimum PDA temperature can be sensitive to the ALD growth temperature. Here, we have shown the implication on photoelectrode performance using TiO_2_/n^+^-Si as a model system, but we suggest that the performance of any Si-based photoelectrode can be improved by decreasing the PDA temperature that can be enabled by careful optimization of the ALD process.

## Conclusions

In summary, we have studied the effect of standard cleaning treatments of Si wafers on the fabrication of the ALD TiO_2_ photoelectrode coating for photoelectrochemical applications. The TiO_2_/Si thin film morphology was quantitatively analyzed using nondestructive XPS measurement. Wet chemical cleaning of Si wafer either by exposing the wafer to dilute HF solution or by boiling an HF-dipped wafer in HCl–H_2_O_2_–H_2_O did not improve the performance of the 30 nm thick TiO_2_ thin film under PEC conditions for water splitting reaction compared to the TiO_2_ film grown on native Si oxide (thickness, *t* = 0.7 nm). Instead, the HF dip cleaning resulted in a hydrophobic oxide-free Si surface (*t* = 0.0 nm) that hindered the initial TiO_2_ growth and the chemical Si oxide (*t* = 0.4 nm) that formed in HCl–H_2_O_2_–H_2_O was nonuniform. The as-deposited TiO_2_ thin films were amorphous and subject to photocorrosion. However, the TiO_2_ thin film was found to be stable over a time period of 10 h at 1.23 V in 1 M NaOH after heat treatment at 400 °C that induced crystallization of amorphous TiO_2_ into anatase TiO_2_. Substrate cleaning prior to the ALD growth did not significantly affect the anatase grain size that was the largest (>10 μm) for the films grown on the native Si oxide. No interfacial Si oxide was formed during the ALD growth, but during the heat treatment, the thickness of interfacial Si oxide increased to 1.8 nm for all of the samples. By increasing the growth temperature from 100 to 150 °C, we were able to reduce the required postdeposition annealing temperature to 300 °C that reduced the formation of interfacial Si oxide and resulted in improved PEC performance.

These results clearly indicate that even though the interfacial Si oxide and the initial ALD process were sensitive to the Si substrate cleaning, the choice of cleaning method had only little effect on the structure and performance of the 30 nm thick TiO_2_ thin film as a photoelectrode protection layer. Further improvement in the performance of ALD TiO_2_/Si photoelectrode would require the development of new precursor chemistry for low-temperature ALD, adjusting the ALD growth parameters, or developing a postgrowth heat treatment of the as-deposited TiO_2_ thin film to result in a crystalline low-defect TiO_2_ structure at a lower temperature, preferably <250 °C, where the formation of interfacial Si oxide that is detrimental to the charge transfer can be limited to an oxide thickness of <1 nm.

## Supporting Information Available

XPS and SEM analyses and data from photoelectrochemical measurements (PDF)
