# Supplementary material for: Interface Engineering of TiO2 Photoelectrode Coatings Grown by Atomic Layer Deposition on Silicon
Source: ACS Omega. 2021 Oct 7;6(41):27501–9. doi: 10.1021/acsomega.1c04478 (PMC8529674; doi:10.1021/acsomega.1c04478)
Supplement: Supplementary file 1 — ao1c04478_si_001.pdf [file ao1c04478_si_001.pdf]

Supporting Information

Interface Engineering of TiO_2 Photoelectrode Coatings Grown by Atomic Layer Deposition on Silicon

Jesse Saari,[,†] Harri Ali-Löytty,[*,†] Mari Honkanen,[‡] Antti Tukiainen,[§] Kimmo Lahtonen,[§] and Mika Valden[*,†]*

[†]*Tampere University, Surface Science Group, Faculty of Engineering and Natural Sciences, P.O.B. 692, Tampere University, FI 33014, Finland*

[‡]*Tampere University, Tampere Microscopy Center, Faculty of Engineering and Natural Sciences, P.O.B. 692, Tampere University, FI 33014, Finland*

[§]*Tampere University, Faculty of Engineering and Natural Sciences, P.O.B. 692, Tampere University, FI 33014, Finland*

X-ray photoelectron spectroscopy analysis of thin film morphology

The X-ray photoelectron spectroscopy was applied to quantitatively analyze the morphology of samples assuming $TiO_2/SiO_2/Si$ overlayer structure. Based on the fundamentals of the electron spectroscopy technique and Beer-Lambert law the XPS intensities of Ti 2p, Si^{4+} 2p and Si^0 2p signals from $TiO_2/SiO_2/Si$ heterostructure are given by

$$I_{\mathrm{TiO2}} = S_{\mathrm{TiO2}}^{\mathrm{Ti\ 2p}} \lambda_{\mathrm{TiO2}}^{\mathrm{Ti\ 2p}} [1 - \exp\left(-t_{\mathrm{TiO2}} / \lambda_{\mathrm{TiO2}}^{\mathrm{Ti\ 2p}}\right)], \quad (1)$$

$$I_{\mathrm{SiO2}} = S_{\mathrm{SiO2}}^{\mathrm{Si4+\ 2p}} \lambda_{\mathrm{SiO2}}^{\mathrm{Si4+\ 2p}} [1 - \exp\left(-t_{\mathrm{SiO2}} / \lambda_{\mathrm{SiO2}}^{\mathrm{Si4+\ 2p}}\right)][\exp\left(-t_{\mathrm{TiO2}} / \lambda_{\mathrm{TiO2}}^{\mathrm{Si4+\ 2p}}\right)], \quad (2)$$

$$I_{\mathrm{Si}} = S_{\mathrm{Si}}^{\mathrm{Si\ 2p}} \lambda_{\mathrm{Si}}^{\mathrm{Si\ 2p}} [\exp\left(-t_{\mathrm{SiO2}} / \lambda_{\mathrm{SiO2}}^{\mathrm{Si\ 2p}}\right)][\exp\left(-t_{\mathrm{TiO2}} / \lambda_{\mathrm{TiO2}}^{\mathrm{Si\ 2p}}\right)], \quad (3)$$

where S_f^j is the photoemission spectroscopy constant for electrons of core level j from film f, λ_f^j the inelastic mean free path (IMFP) of electrons of core level j attenuated by film f, and t_f the thickness of the film f[1]. The photoemission spectroscopy constant consists of factors as follows

$$S_f^j = \Phi_f(h\nu) \times \sigma_f^j(h\nu) \times \beta_f^j(h\nu) \times D_f(KE) \times N_f, \quad (4)$$

where Φ is the X-ray flux, σ the cross section, β the asymmetry parameter, D the spectrometer efficiency and N the number of atoms per unit volume[1]. The thickness of the interfacial SiO_2 can be derived from the ratio $\frac{I_{\mathrm{SiO2}}}{I_{\mathrm{Si}}}$ as follows

$$t_{\mathrm{SiO2}} = \lambda_{\mathrm{SiO2}}^{\mathrm{Si\ 2p}} \ln\left[1 + \frac{I_{\mathrm{SiO2}}}{I_{\mathrm{Si}}} \frac{N_{\mathrm{Si}} \lambda_{\mathrm{Si}}^{\mathrm{Si\ 2p}}}{N_{\mathrm{SiO2}} \lambda_{\mathrm{SiO2}}^{\mathrm{Si4+\ 2p}}}\right], \quad (5)$$

by approximating $\lambda_{\mathrm{SiO2}}^{\mathrm{Si\ 2p}} = \lambda_{\mathrm{SiO2}}^{\mathrm{Si4+\ 2p}}$ and canceling out Φ, σ, β and D because of the electron energy analyzer magic angle (54.7°)[2], the use of the Scofield photoionization cross-sections[3] as relative sensitivity factors and the almost equal kinetic energy of the Si^0 2p and Si^{4+} 2p core level electrons. Analogously, but without the approximation of $\lambda_{\mathrm{TiO2}}^{\mathrm{Ti\ 2p}} = \lambda_{\mathrm{TiO2}}^{\mathrm{Si4+\ 2p}}$ due to the kinetic energy difference of the Ti 2p and Si^{4+} 2p core level electrons, the thickness of TiO_2 can be determined by solving the following equation

$$\frac{I_{\mathrm{TiO2}}}{I_{\mathrm{SiO2}}}\frac{N_{\mathrm{SiO2}}\lambda_{\mathrm{SiO2}}^{\mathrm{Si4+\,2p}}}{N_{\mathrm{TiO2}}\lambda_{\mathrm{TiO2}}^{\mathrm{Ti\,2p}}}\left[1-\exp\left(-t_{\mathrm{SiO2}}/\lambda_{\mathrm{SiO2}}^{\mathrm{Si4+\,2p}}\right)\right]+\exp\left[t_{\mathrm{TiO2}}\left(1/\lambda_{\mathrm{TiO2}}^{\mathrm{Si4+\,2p}}-1/\lambda_{\mathrm{TiO2}}^{\mathrm{Ti\,2p}}\right)\right]-\exp\left[t_{\mathrm{TiO2}}/\lambda_{\mathrm{TiO2}}^{\mathrm{Si4+\,2p}}\right]=0. \qquad (6)$$

For the as-deposited TiO_2 on HF-treated substrates which show no interfacial SiO_2 layer the above written equation changes as follows

$$\frac{I_{\mathrm{TiO2}}}{I_{\mathrm{Si}}}\frac{N_{\mathrm{Si}}\lambda_{\mathrm{Si}}^{\mathrm{Si\,2p}}}{N_{\mathrm{TiO2}}\lambda_{\mathrm{TiO2}}^{\mathrm{Ti\,2p}}}+\exp\left[t_{\mathrm{TiO2}}\left(1/\lambda_{\mathrm{TiO2}}^{\mathrm{Si\,2p}}-1/\lambda_{\mathrm{TiO2}}^{\mathrm{Ti\,2p}}\right)\right]-\exp\left[t_{\mathrm{TiO2}}/\lambda_{\mathrm{TiO2}}^{\mathrm{Si\,2p}}\right]=0. \qquad (7)$$

The IMFP values of $\lambda_{\mathrm{Si}}^{\mathrm{Si\,2p}} = 3.09$ nm, $\lambda_{\mathrm{SiO2}}^{\mathrm{Si\,2p}} = \lambda_{\mathrm{SiO2}}^{\mathrm{Si4+\,2p}} = 3.75$ nm, $\lambda_{\mathrm{TiO2}}^{\mathrm{Si\,2p}} = \lambda_{\mathrm{TiO2}}^{\mathrm{Si4+\,2p}} = 2.67$ nm and $\lambda_{\mathrm{TiO2}}^{\mathrm{Ti\,2p}} = 2.12$ nm were calculated from TPP-2M formula[4]. The number of atoms per unit volume values used in the calculations were $N_{\mathrm{Si}} = 49.737\ \mathrm{atoms/nm^3}$, $N_{\mathrm{SiO2}} = 26.557\ \mathrm{atoms/nm^3}$ and $N_{\mathrm{TiO2}} = 31.900\ \mathrm{atoms/nm^3}$. This derivation assumes only inelastic scattering of photoelectrons. An error resulting from neglecting elastic scattering was determined using SESSA simulation software[5] to be <10% for the reported values.

Table S1. The relative concentrations of the components (at.%) on as-received native oxide, HF-treated and HF + RCA SC-2 -treated Si(100) surfaces measured by XPS.

Relative concentrations of components (at.%)						
	**C**			**O**	**Si**	
	C-C/H	C-O	(O-)C=O	O	Si^0	Si^{4+}
Native oxide Si	2.91	1.45	0.49	27.49	59.69	7.95
HF-treated Si	3.19	1.13	–	4.27	91.41	–
HF+RCA-2 Si	3.86	1.13	0.48	21.83	67.92	4.78

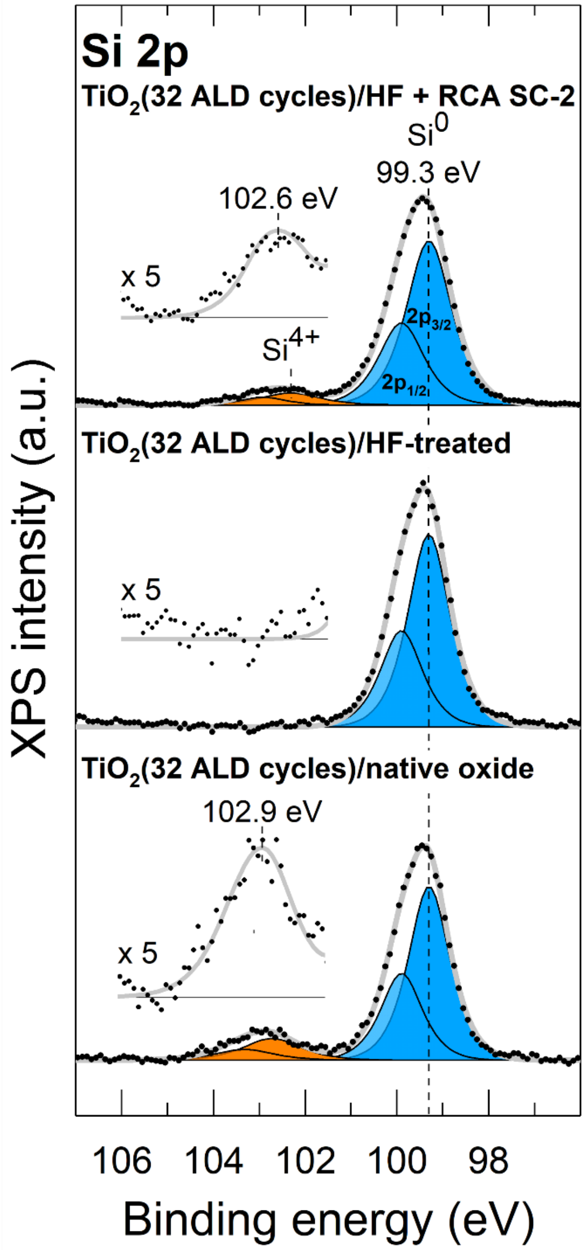

Figure S1. The XP Si 2p spectra showing the interfacial SiO_2 layer of the TiO_2/Si(native oxide), TiO_2/Si(HF-treated) and TiO_2/Si(HF + RCA SC-2) interfaces after 32 ALD TiO_2 cycles.

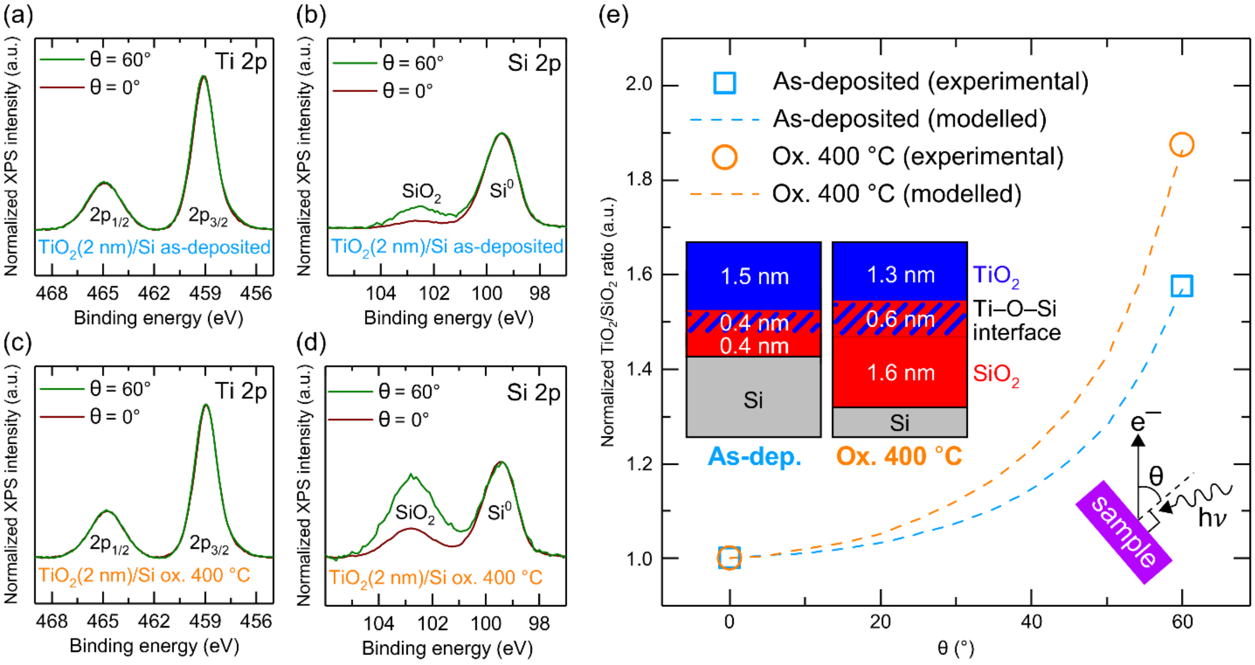

Figure S2. Angle resolved XPS analysis for ALD TiO_2(2 nm) thin film grown on HF + RCA SC-2 -treated Si(100). Ti 2p (a, c) and Si 2p (b, d) XP spectra recorded after deposition (a, b) and after oxidation at 400 °C (c, d) at two electron emission angles (0° and 60°). (e) TiO_2/SiO_2 atomic ratio as a function of electron emission angle normalized to the value obtained at 0°. Modelled oxide layer morphologies are shown as insets in (e). The modelled results (dashed lines) were obtained using SESSA simulation software and TiO_2/$TiSiO_2$/SiO_2/Si layer structure[5].

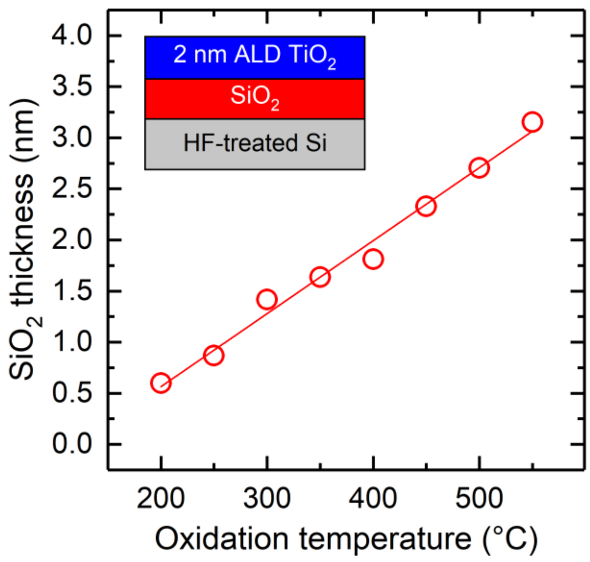

Figure S3. Thickness of interfacial Si oxide as a function of oxidation temperature 200–550 °C for ALD TiO_2(2 nm) grown on oxide free Si(100) substrate analyzed based on photoelectron signal attenuation according to the Beer–Lambert law assuming homogeneous overlayer (Eq. 6).

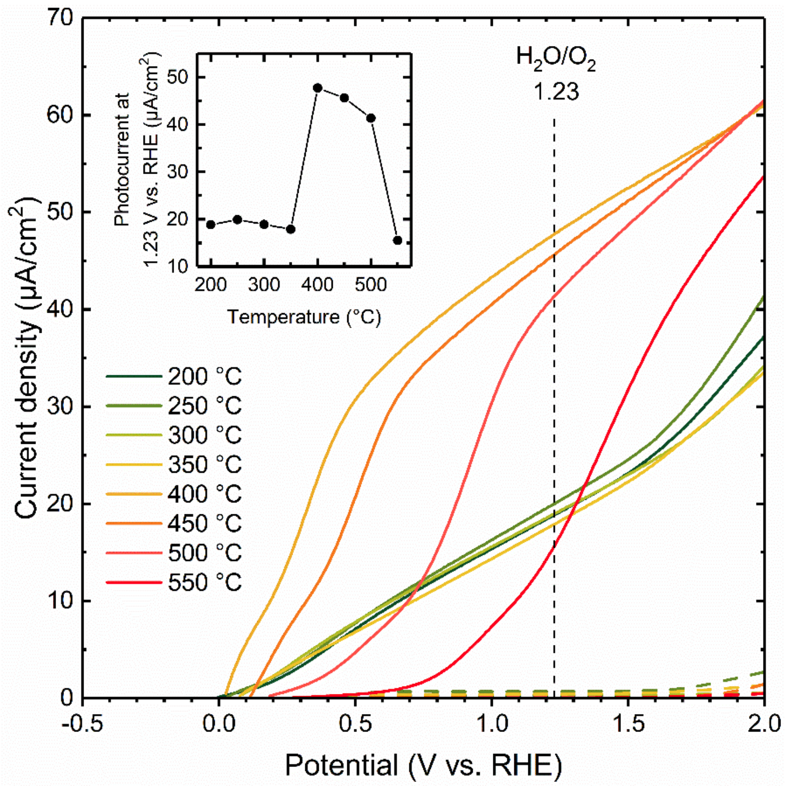

Figure S4. The current-voltage characteristics in dark (dashed lines) and under simulated sunlight (solid lines) measured in 1 M NaOH by linear sweep voltammetry for ALD TiO_2(30 nm, 100 °C)/ n^+-Si photoanodes heat treated at different temperatures. The inset highlights that the maximum photocurrent for H_2O oxidation was obtained after the heat treatment at 400 °C, i.e, after crystallization of am.-TiO_2. For higher heat treatment temperatures, the photocurrent decreases due to the increasing thickness of electrically resistive interfacial Si oxide.

Table S2. The XPS results. The relative concentrations of elements on as-deposited and ox. 400 °C ALD TiO_2(30 nm, 100 °C) grown on as-received native oxide, HF-treated and HF + RCA SC-2 -treated Si(100).

Relative concentrations of elements (at.%)				
	**C**	**O**	**Ti**	**N**
TiO_2(30 nm, 100 °C)/native oxide as-deposited	12.99	59.95	25.73	1.32
TiO_2(30 nm, 100 °C)/HF-treated as-deposited	13.42	59.92	25.62	1.05
TiO_2(30 nm, 100 °C)/HF + RCA SC-2 as-deposited	12.39	60.83	25.75	1.03
TiO_2(30 nm, 100 °C)/native oxide ox. 400 °C	11.41	61.58	26.64	0.36
TiO_2(30 nm, 100 °C)/HF-treated ox. 400 °C	10.05	62.61	26.48	0.43
TiO_2(30 nm, 100 °C)/HF + RCA SC-2 ox. 400 °C	10.61	61.93	27.07	0.39

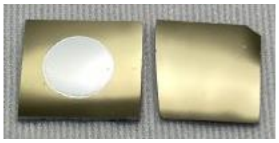

Figure S5. Photo of as-deposited (left) and heat-treated at 400 °C (right) ALD TiO_2(30 nm, 100 °C)/n^+-Si photoanodes after 10 h stability test under 1 sun illumination at 1.23 V vs. RHE in 1 M NaOH. As-deposited amorphous TiO_2 coating has dissolved completely from the circular area exposed to the electrolyte. Heat-treated anatase TiO_2 coating does not show visual degradation.

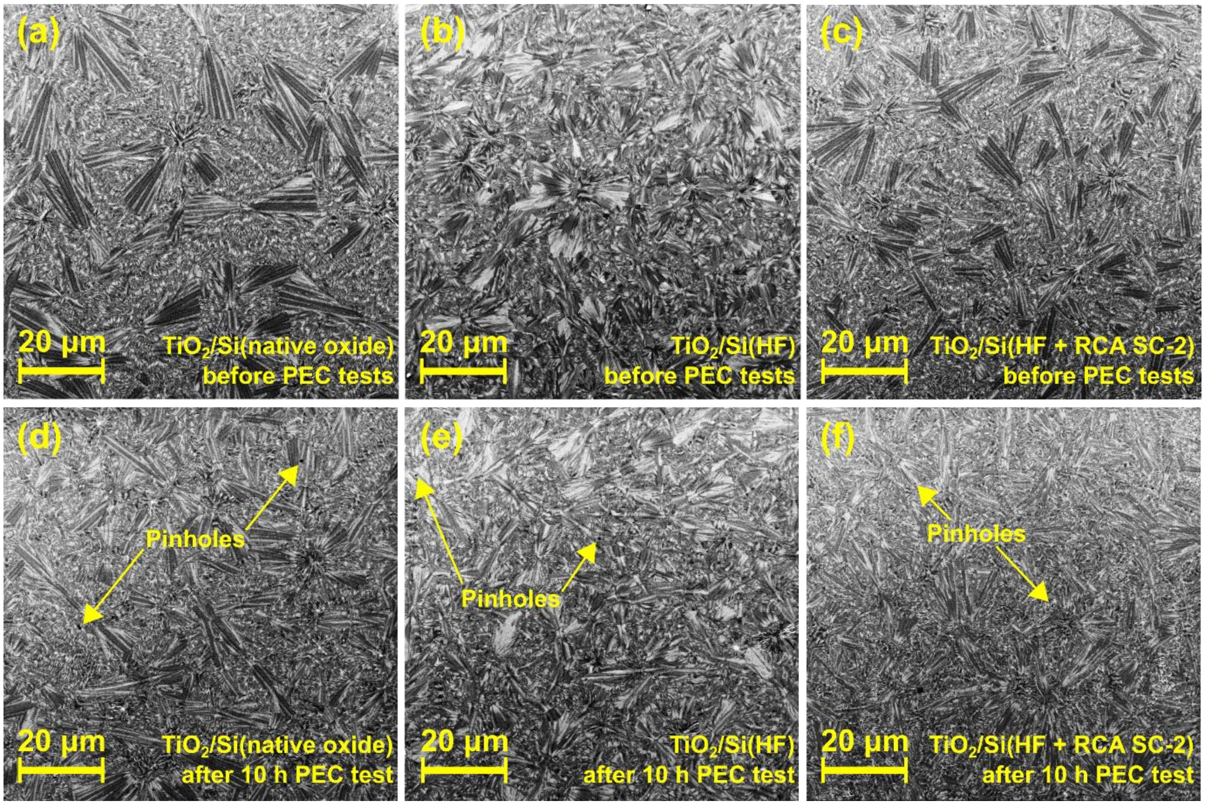

Figure S6. SEM analysis of 400 °C oxidized (anatase) TiO_2 thin films on n^+-Si substrates with different surface treatments before (a–c) and after (d–f) 10 h stability test at 1.23 V vs. RHE in 1 M NaOH under 1 Sun. Few pinholes were detected in all the samples after the stability test.

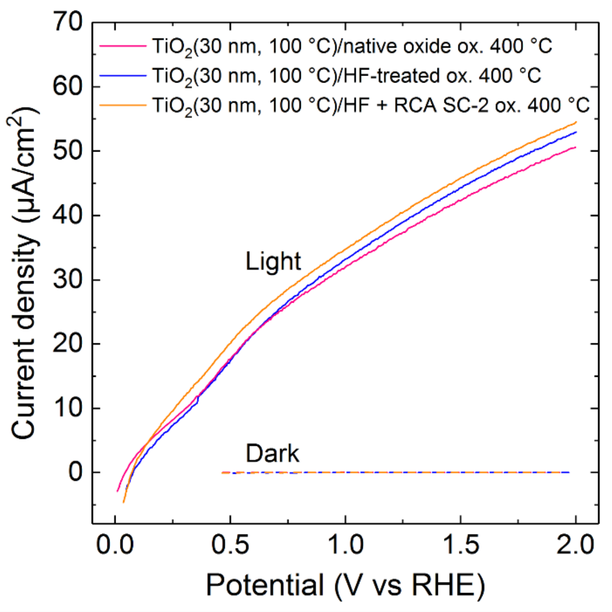

Figure S7. The current-voltage characteristics in dark (dashed lines) and under simulated solar illumination (solid lines) measured in 1 M NaOH by linear sweep voltammetry for ALD TiO_2(30 nm) thin films grown at 100 °C on n$^+$-Si with different surface treatments and subsequently oxidized at 400 °C.

REFERENCES

(1) Newberg, J. T.; Starr, D. E.; Yamamoto, S.; Kaya, S.; Kendelewicz, T.; Mysak, E. R.; Porsgaard, S.; Salmeron, M. B.; Brown, G. E.; Nilsson, A.; Bluhm, H. Formation of Hydroxyl and Water Layers on MgO Films Studied with Ambient Pressure XPS. *Surface Science* **2011**, *605* (1–2), 89–94. https://doi.org/10.1016/j.susc.2010.10.004.

(2) Reilman, R. F.; Msezane, A.; Manson, S. T. Relative Intensities in Photoelectron Spectroscopy of Atoms and Molecules. *Journal of Electron Spectroscopy and Related Phenomena* **1976**, *8* (5), 389–394. https://doi.org/10.1016/0368-2048(76)80025-4.

(3) Scofield, J. H. Hartree-Slater Subshell Photoionization Cross-Sections at 1254 and 1487 eV. *Journal of Electron Spectroscopy and Related Phenomena* **1976**, *8* (2), 129–137. https://doi.org/10.1016/0368-2048(76)80015-1.

(4) QUASES-IMFP-TPP2M - QUASES http://www.quases.com/products/quases-imfp-tpp2m/ (accessed 2020 -06 -26).

(5) Powell, C. J. NIST Database for the Simulation of Electron Spectra for Surface Analysis (SESSA), Version 2.1.1. **2018**.
